# Advances in understanding and treating persecutory delusions: a review

**DOI:** 10.1007/s00127-014-0928-7

**Published:** 2014-07-09

**Authors:** Daniel Freeman, Philippa Garety

**Affiliations:** 1 Department of Psychiatry, University of Oxford, Warneford Hospital, Oxford, OX3 7JX UK; 2 King’s College London, London, UK

**Keywords:** Delusions, Persecutory, Schizophrenia, Psychosis, Paranoia

## Abstract

**Purpose:**

Persecutory delusions are a central psychotic experience, at the severe end of a paranoia spectrum in the general population. The aim of the review is to provide an introduction to the understanding of persecutory delusions, highlight key putative causal factors that have the potential to be translated into efficacious treatment, and indicate future research directions.

**Methods:**

A narrative literature review was undertaken to highlight the main recent areas of empirical study concerning non-clinical and clinical paranoia.

**Results:**

Six main proximal causal factors are identified: a worry thinking style, negative beliefs about the self, interpersonal sensitivity, sleep disturbance, anomalous internal experience, and reasoning biases. Each has plausible mechanistic links to the occurrence of paranoia. These causal factors may be influenced by a number of social circumstances, including adverse events, illicit drug use, and urban environments.

**Conclusions:**

There have been numerous replicated empirical findings leading to a significant advance in the understanding of persecutory delusions, now beginning to be translated into cognitive treatments. The first trials specifically focussed on patients who have persecutory delusions in the context of psychotic diagnoses are occurring. Initial evidence of efficacy is very promising.

## Introduction

‘My neighbours are spreading nasty rumours and are tormenting me’

‘MI5, MOSSAD, and the police are trying to get me and torture me’

‘An evil spirit is out to kill me’ [1]

Two distressing concerns are at the heart of persecutory delusions: harm is going to occur and others intend it [2]. Almost half of individuals with persecutory delusions have levels of psychological well-being in the lowest 2 % of the general population [3]. The delusions are typically accompanied by anxiety [4], depression [5], and disturbed sleep [6]. A personal account by Weiner [7] notes: ‘What I remember most is how disoriented and frightened I felt.’ At first episode of psychosis, over 70 % of patients have a persecutory delusion [8, 9]. It is the type of delusion most likely to be acted upon [10]. Persecutory delusions are a common, clinically important, psychotic experience, for which treatments need to be significantly improved. We advocate the approach of understanding the causes to translate this knowledge into efficacious treatment [11].

**Fig. 1:**
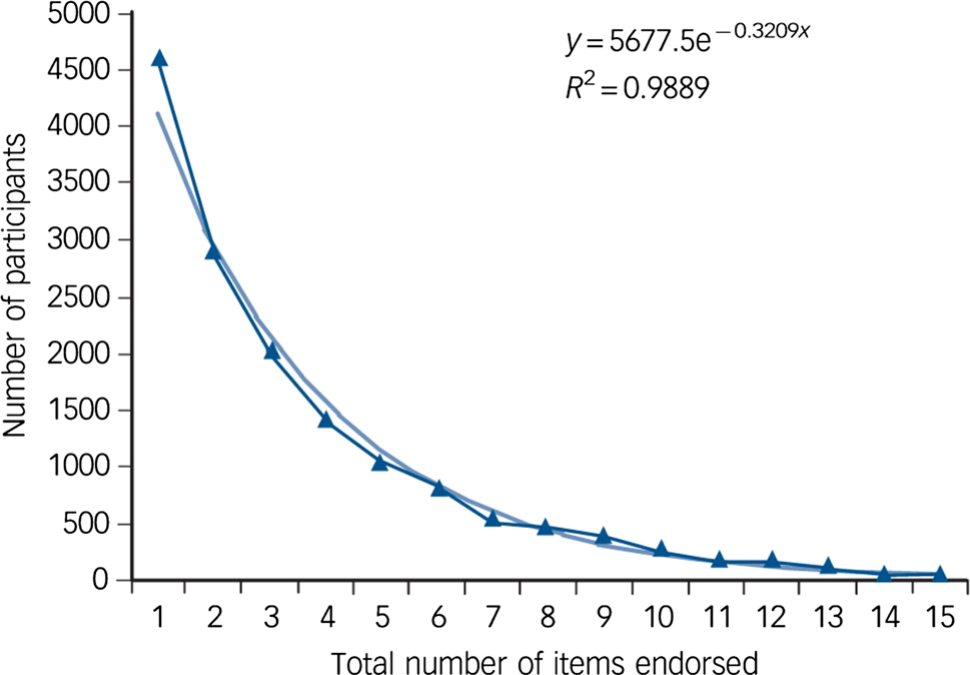
The distribution of total paranoia scores in the general population [15]

Increasingly it is being recognised that persecutory delusions are at the extreme end of a paranoia spectrum. It is another example, more widely accepted for common mental health problems, of a quantitative trait in the general population [12, 13]. As illustrated in Fig. 1, there is an exponential distribution of paranoid thoughts in the general population [14, 15]. This distribution of paranoia has even been found in children [16]. Many people have a few paranoid thoughts, and a few have many. Paranoia in the general population is associated with poorer physical health, suicidal ideation, and weaker social cohesion [17]. Only recently has the heritability of paranoia in the general population been estimated. In a study of five thousand adolescent twin pairs, it was found that 50 % of the variability in levels of paranoia in the population is due to genes [18]. Identifying the genes is likely to prove difficult [19], though of course the heritability estimate indicates that the environment has an equally important role in the occurrence of paranoia. Paranoia in adulthood generally decreases slightly with age, and though there may be content differences the rates appear similar in men and women [17].

Study of individual psychotic experiences has gained ground because of the evidence that the main diagnoses of psychosis, such as schizophrenia, schizo-affective disorder, and delusional disorder, do not capture single disorders. Despite their longevity of use, the diagnoses may prove an obstacle in the advancement of the understanding and treatment of the difficult experiences for which patients require help. The empirical research indicates that within these diagnoses are multiple independent experiences, such as paranoia, hallucinations, grandiosity, thought disorder, and anhedonia [13, 20–22]. A research approach, particularly adopted by cognitive psychology, has been to try to explain these single psychotic experiences. We note that most causes are ‘inus conditions’—‘an *insufficient* but *non*-*redundant* part of an *unnecessary* but *sufficient* condition’ [23]. Paranoia arises from a combination of causes, with each causal factor only increasing the probability of such fears occurring. This review highlights our perspective on the established findings, the most promising directions, and key research questions. The empirical evidence here is synthesised from an updated search continuing on from our three earlier systematic literature reviews of delusions [24–26].

## A worry thinking style

‘… sit and think. Then get paranoider and paranoider and paranoider and paranoider’ [27]

Worry brings implausible ideas to mind, keeps them there, and increases the distress that they cause. It is therefore a plausible factor in the occurrence of paranoid thinking [28]. The evidence to support this position has been accumulating. A longitudinal national epidemiological survey showed that the presence of worry predicts new inceptions of paranoid thoughts over 18 months [29]. Worry predicts the persistence of existing non-clinical paranoia [29, 30]. Those who tend to adopt a worry thinking style are also more likely to experience paranoia in an experimental setting [31, 32]. Rates of worry in patients with persecutory delusions are comparable to those seen in patients with generalised anxiety disorder [28, 33–35]. Levels of rumination are also high in patients with persistent persecutory delusions [36]. An experience sampling study has shown that a period of worry precedes the occurrence of delusional ideation [37]. Importantly, the level of worry in patients predicts the persistence of persecutory delusions over the following months [5, 38].

These theoretical studies led to two pilot clinical trials that attempted to reduce levels of worry in patients with persecutory delusions. The worry interventions address the tendency to react to troubling thoughts with worry and do not dispute the content of persecutory delusions. The initial evidence was encouraging, indicating that reducing worry may lead to reductions in the delusions [39, 40]. This has now been rigorously tested by Freeman and colleagues [40] in the Worry Intervention Trial, the first major randomised controlled trial specifically for patients with persecutory delusions. 150 patients with persistent persecutory delusions were randomised to a six session worry reduction intervention in addition to standard care or to standard care. Assessments were carried out blind and the follow-up rate was very high. The CBT for worry intervention led to significant reductions in worry and the persecutory delusions. Changes in worry mediated the majority of the change in the delusions. There were also significant improvements in well-being, and reductions in rumination, overall psychiatric symptoms and general levels of paranoia. This is convincing evidence for the importance of a worry thinking style causing the persistence of persecutory delusions and the strongest demonstration to date of the advances in understanding being translated into treatment. Treatment refinement is likely to benefit from studies that determine the mechanisms underlying worry in patients with delusions.

## Negative thoughts about the self

‘After university, however, I failed to really get a good start to my career, I was working part time and living with my parents, I had no clue about what I really wanted to do, and because I had no money, I couldn’t go out very much… I began to be under the impression that I had some sort of social handicap… Eventually, I was convinced that when I was out on the street, everyone who saw me instantly knew I had some sort of social handicap. It actually started to feel as if everybody who met me pretended to treat me normally and then laughed at me behind my back once I’d gone’ [41].

**Fig. 2:**
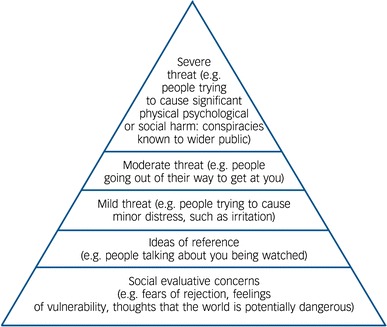
The paranoia hierarchy [14]

The view encapsulated in the paranoia hierarchy (see Fig. 2) [14] is that feeling negative about the self can lead to feelings of being different, apart, inferior and hence vulnerable. Paranoia is likely to flourish when an individual perceives him or herself as potentially vulnerable. Two longitudinal patient studies have shown that negative thoughts about the self predict the persistence of persecutory delusions [5, 42]. In the largest of these, 301 patients with psychosis were assessed three times over a year [42]. Structural equation modelling indicated that negative cognition led to paranoid thinking, with little evidence for the reverse direction. Three recent systematic reviews indicate that paranoia is associated directly with negative self-concepts, without the need to evoke defensive processes [26, 43, 44]. Individuals with persecutory delusions may actually be excessively critical of themselves [45]. A recent experimental study manipulated self-esteem in people vulnerable to paranoid thoughts [46]. The participants entered a virtual social world twice: once at their normal height, once at a reduced height. Height is associated with social status, so it was predicted that reducing height would lead to lower self-esteem. It was found that reducing height led to more negative thoughts about the self in relation to others and this explained an increase in paranoia. Another experimental study has indicated that a compassion focussed technique reduces the occurrence of paranoid ideation during recall of a distressing memory [47].

The evidence for an association of persecutory delusions with negative self-thoughts is convincing, and is consistent with broader work showing links of negative emotion to positive symptoms of psychosis [4] including paranoia [48, 49] and with the ‘social defeat’ hypothesis of schizophrenia [50]. It may also link to the well-established association of living in urban areas with a greater rate of psychotic experiences [51, 52]. In a recent study, we assessed patients with persecutory delusions on a battery of psychological processes before and after being randomised to either entering a busy social urban environment or staying in-doors [53]. Going outside led to a significant increase in paranoia, anxiety, depression, negative thoughts about the self, and fewer positive thoughts about the self. The increase in paranoia was partially mediated by the increases in anxiety, depression, and negative thoughts about the self. This study provides further evidence that negative affect and related processes lead to an increase in paranoia, and indicates one route via which urban environments may have an impact on psychological health.

The findings have been used to develop treatment for the common problem for patients with persecutory delusions of going outside into busy places, tested in a recent case series with fifteen patients [54]. The clear treatment implication of this work is that reducing negative thoughts about the self in patients with persecutory delusions will lead to a lessening of paranoia. In studies that have treated self-esteem and measured psychotic experiences, a reduction in delusions and hallucinations has been seen [55, 56]. These studies have not, however, examined paranoia in particular. Studies testing clinical techniques to improve negative beliefs about the self in patients with persecutory delusions are clearly indicated [57].

## Interpersonal sensitivity

‘Yeh, yeh and that’s the thing, I didn’t realise that it was paranoia but I was terrified that people would laugh at me or ridicule me or hurt me or trick me in some way, you know, mind games became an active part of my perception’ [58]

The exact nature of negative self-cognition in paranoia remains to be determined. Assuredly, such thoughts will be related to the anxiety and depression commonly found in individuals with paranoid ideation. And negative cognitions about the self and others will be one route via which adverse events lead to paranoia [29, 59, 60]. One promising more refined specification is interpersonal sensitivity, defined as ‘feeling vulnerable in the presence of others due to the expectation of criticism or rejection’ [61]. Paranoid ideation can be considered as an extension of such concerns. Interpersonal sensitivity was first linked to paranoia in a series of virtual reality experimental studies [31, 62, 63]. For instance, a study of 200 members of the general population found that people higher in interpersonal sensitivity were more likely to interpret a neutral virtual reality social environment as containing hostility from others [31]. Subsequent work has found interpersonal sensitivity to be high in patients with persecutory delusions [35] and, latterly, in those at high risk of developing psychosis [64]. Interpersonal sensitivity is positively associated with levels of anxiety and depression [65]. Longitudinal studies of interpersonal sensitivity have yet to be carried out.

A recent pilot study has provided an initial test of the effect on persecutory delusions of intervening on interpersonal sensitivity [61]. Eleven patients with persistent persecutory delusions and reporting interpersonal sensitivity took part. There was a baseline period of a fortnight, provision of six sessions of CBT focussed upon reducing interpersonal sensitivity cognitions, and a 1-month follow-up. Patients were stable during the baseline period but following the intervention there were large effect size reductions in both interpersonal sensitivity and the persecutory delusions which were maintained at the follow-up. However, assessments were carried out by the therapist, and there was no control group. Targeting interpersonal sensitivity in patients with persecutory delusions requires evaluation in a randomised controlled study. There is scope for greater precision in the measurement of the concept. More broadly, a topic needing sustained research is specification of the negative cognitions central to paranoia, clarifying whether there are a variety of types of negative cognitions about the self and others; qualitative surveys may be of particular help here initially. The most proximal ideation to paranoia is likely to concern vulnerability, which may stem from a number of different views about the self and others.

## Anomalous internal experiences

‘It isn’t as much the dark that I’m afraid of now, it’s the feeling of what may be in the room that I cannot see. I always feel like someone is there, and is going to either kidnap, rape, or kill me. Many times when I am home alone I feel that someone is going to break in and kill me. I always feel that someone is there’ [66].

In the study of paranoia there is often an emphasis given to the misinterpretation of external events; in the attributional literature, this is typically about the occurrence of outright negative events, such as a friend being hostile [67]. Clinically, the events reported by patients are actually mostly minor and ambiguous, such as someone bumping into the person, or the looks on faces, or snippets of overheard conversations. However, it is often under-appreciated that these events are common in the environment and are not always noticed or misinterpreted by patients. An alternative view is that these events only gain significance when the individual is in a subjectively unsettled state. The implication is that what is occurring is actually a misinterpretation of internal anomalous experience. The person experiences, for example, unexplained anxious arousal, or feelings of depersonalisation, or has perceptual disturbances, which go unrecognised and lead to explanations sought in the external environment. Thus, external events are fuel for the misinterpretations of the internal states.

Such anomalous experiences may be triggered by illicit drugs, poor sleep, and negative life events. There is even recent evidence that a bout of worry can cause depersonalisation in patients with persecutory delusions [68]. There are many reports of a wide-range of anomalous internal experiences in patients with psychosis [69–72]. One long-standing framing of these anomalous states in patients has been the idea that there are basic self disturbances in schizophrenia [73], and another that there is aberrant salience [74]. Those vulnerable to anomalous internal experiences are more likely to experience paranoia in an experimental setting [75]. The account of delusions arising from changes in subjective experience is also used to explain an association of hearing loss and psychotic experiences [76], which was tested in one of the first experimental studies of paranoia [77]. The appraisals of the anomalous experiences contribute to the level of distress caused [78, 79].

The creation of internal subjective states in experimental tests of paranoia is rare. A recent study used the intravenous administration of ∆^9^-tetrahydrocannabinol (THC), the principal psychoactive ingredient of cannabis, to test a causal role for anomalous experiences in paranoia [80]. A randomised, placebo-controlled test with 121 individuals from the general population reporting paranoid ideation showed that administration of THC caused the occurrence of paranoia, as assessed by virtual reality, self-report, and semi-structured interview. The THC also caused an increase in anomalous internal experiences and negative affect and a decrease in working memory performance. It was the increase in anomalous experience and negative affect, and not the changes in working memory, that fully mediated the increase in paranoia. Interestingly, it was not possible to disentangle the separate contributions of anomalous experiences and negative emotion, indicating common connections. Of importance now are studies that help reduce anomalous internal experiences in patients with persecutory delusions.

## Insomnia

‘I’ve even been too scared while driving to look in the rear view mirror because I knew I would ‘see’ the bloke who’s there ready to kill me. I wouldn’t sleep for hours and hours because I thought as soon as I closed my eyes there would be someone standing there when I opened them’ [66].

An area of expansion in the past 5 years has been the role of sleep disturbance in the occurrence of paranoia. Indeed, sleep as a causal factor across psychiatric problems is receiving greater attention [81, 82]. The obvious routes by which problems sleeping could lead to paranoia are an increase in negative affect and in the occurrence of subtle anomalies of experience. The first systematic report on the issue found high rates of insomnia in patients with persecutory delusions [6]. Subsequent studies found substantial associations of paranoid ideation with insomnia in the general population, which were partially mediated by negative affect [17, 83]. A longitudinal population study showed that having insomnia increased the odds by threefold of developing paranoid ideation [29]. Poor sleep has also been found to be a predictor of the persistence of existing paranoia [30]. Supporting these ideas of important causal connections between disturbed sleep and paranoia, a study of over 5,000 adolescent twin pairs found paranoia and insomnia to be associated and that there is significant overlap between the two problems in genetic and environmental risk [84].

Given that insomnia is a treatable condition [85, 86], there is an obvious potential for translation of this theoretical knowledge about the contribution of sleep to paranoia. In the only study to date, 15 patients with persistent persecutory delusions all received a four-session session CBT intervention for insomnia [87]. There were large effect size reductions in both insomnia and the delusions, which persisted at least 1 month after intervention. However, this was an unblinded, uncontrolled study. A longer CBT for insomnia intervention for patients with delusions and/or hallucinations is now being evaluated in a randomised controlled clinical trial [88]. Of course if patients with psychosis have sleep problems then these should be treated with the available evidence-based interventions, but the implication is that this will also lessen the psychotic experiences. Identification of the factors, both proximal and distal, leading to chronic sleep problems in patients with delusions would be beneficial for treatment development.

## Reasoning

‘When I see people laughing and talking about me I try thinking that I’m jumping to conclusions. When I see people with mobiles I’ve been trying to give them the benefit of the doubt—they might be taking pictures of me, but they might not. It’s less distressing thinking like this…’ [89]

One of the most replicated findings is the presence of ‘jumping to conclusions’ (JTC) being more common in patients with delusions than in non-clinical populations [90–92]. These studies have typically contained a majority of patients with persecutory delusions, so we can be confident that the bias is present in this delusion subtype although rates of JTC may be even higher in patients with grandiose delusions [93]. Jumping to conclusions, reaching certainty after limited data gathering, is considered to lead to the rapid acceptance of delusional ideas. Jumping to conclusions is associated with lower working memory capacity [94, 95], but not higher levels of need for closure [96] or intolerance of uncertainty [95]. It is present in high conviction delusional beliefs but not in high conviction anxiety beliefs [97]. In clinical groups the presence of the bias does not seem to be affected by anxiety manipulations [68, 98].

Standard assessments of JTC do not examine the type of data gathered. However, the gathering of data is highly likely to be influenced by the well-established belief confirmation bias [99], or a bias against disconfirmatory evidence [100]. Once data are gathered, the influence of experiential and analytic reasoning may be important. In the general population, higher levels of paranoia are associated with less use of analytic reasoning [101, 102]. Individuals with persecutory delusions report less use of both experiential and rational reasoning styles [102]. JTC, belief confirmation, and less use of analytic reasoning are all likely to lead not only to a strongly held delusional belief but the failure to consider alternative explanations. A failure to consider alternative explanations [103], resistance to hypothetical contradiction [10], and an unwillingness to consider the possibility of being mistaken [10] are all considered as markers of ‘belief inflexibility’ [104]. Belief inflexibility is the reasoning process most associated with degree of conviction in delusions [105].

**Fig. 3:**
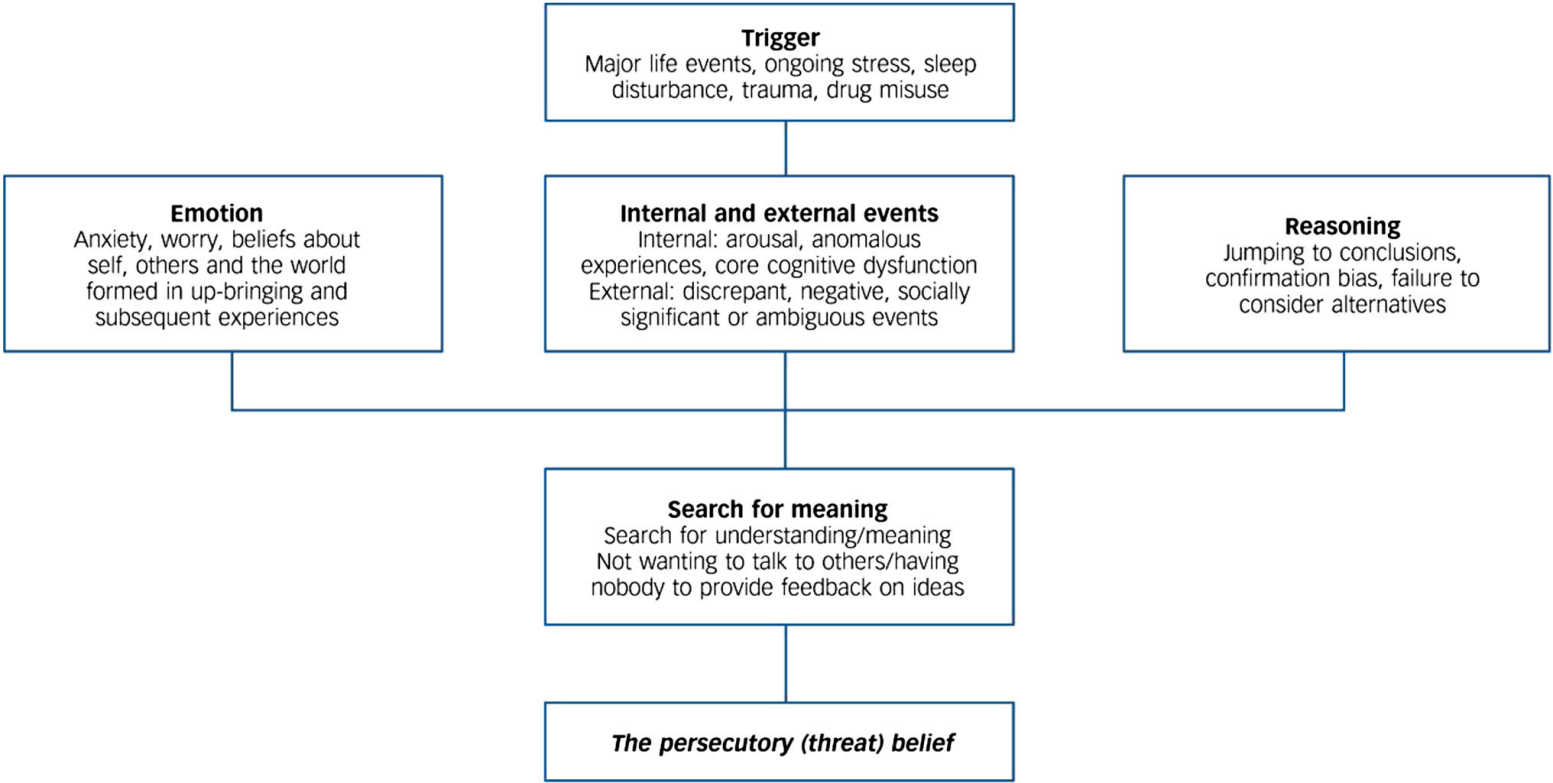
Outline of factors involved in delusion formation [31]

Attention has turned towards how this knowledge of reasoning processes in delusions can be translated into treatment. A randomised controlled trial with 154 patients with paranoia showed that a novel educational approach (metacognitive training, MCT), providing information and exercises about a number of cognitive biases found in psychosis, given within groups over eight sessions, did not have an impact on delusions [106]. A similar-sized trial suggested that MCT had benefits for patients without severe clinical delusions, i.e. with mild to moderate levels of delusional ideation [107]. These have been the two most rigorous, well-powered tests of this group reasoning educational programme, showing contrasting results. Building on this innovative work, our approach in working with people with high conviction delusions has been first to demonstrate in experimental studies short-term change in the reasoning biases [89, 108]. In a recent study, 101 patients with current delusions were randomised to a 90-min individually delivered reasoning intervention or to an attention control condition [109]. There were homework exercises over the following fortnight for patients receiving the intervention. The brief reasoning training led to significant reductions in state paranoia and improvements in reasoning: reduced JTC and increased belief flexibility. There was evidence that the increases in belief flexibility partially mediated a significant reduction in current levels of paranoia in the patients at the 2-week assessment. The study demonstrates that it is possible, at least in the short-term, to help people adjust their reasoning styles, and that this leads to change in paranoia. Belief flexibility is therefore a promising reasoning target. The next step is the development and evaluation of a longer, eight session clinical intervention aiming to achieve effective flexibility in thinking and consequent sustained change in the delusion; this is a key area for future research. Overall, the findings in our review indicate the importance of affective processes, anomalous experiences, and reasoning in the occurrence of severe paranoia (see Fig. 3).

### Other paths

Perhaps the most researched psychological process in schizophrenia research has been theory of mind [110], since almost by definition the intentions of others are being misread in the content of paranoid thoughts. There is strong evidence for theory of mind difficulties being present in patients with schizophrenia [111, 112]; however, it is clear that these cognitive difficulties are most associated with negative symptoms and not paranoia [25, 26, 113]. In our recent review, in 38 clinical studies of theory of mind, the majority did not find associations with delusions in general or paranoia [26]. In three non-clinical studies there was no association of theory of mind performance and paranoia. A recent study with patients with an early episode of psychosis and non-clinical controls found no association of a dimensional paranoia measure with theory of mind performance in either group [114]. Overall, this is unsurprising. It is known that theory of mind problems is closely linked to cognitive neuropsychological impairments [115, 116] and that such cognitive impairments are linked with the negative rather than the positive symptoms of psychosis such as delusions [117]—the theory of mind results in schizophrenia is consistent with this pattern. Furthermore, an interesting analysis of patient conversations found theory of mind skills to be intact [118]. Our view is that in clinical settings it is possible that theory of mind difficulties is present in some patients, that when present they could exacerbate paranoid thinking, but such problems are not a key causal factor.

A topic that has gained attention but received less direct empirical scrutiny has been the hypothesis of two distinct types of paranoia: ‘Poor me’ and ‘bad me’ [119]. In the former paranoia is considered a defence against negative emotions reaching consciousness and in the latter paranoia is considered a direct reflection of conscious ideas about the self that are so extremely negative that the person believes they will be punished. In clinical populations ‘Poor Me’ presentations are substantially more common than ‘Bad Me’ [120–122]. The plausibility of there being two distinct types of paranoia with opposite causes is debateable; indeed, analysis of an epidemiological national survey indicates there to be just a single underlying paranoia dimension [15]. Importantly, the central test of the two subtypes of hypothesis has not been carried out: an examination of defence processes in each of the two types. Given that the Poor Me subtype is substantially more common, it is therefore notable that the evidence is generally inconsistent with a defence account of persecutory delusions: hypothesised defences are hard to test but the largest studies [123, 124] do not find defensive processing present; defensive processing has not even been found for the more obviously self-enhancing grandiose delusions [125]; while systematic literature reviews have all reported a strong direct relationship between negative emotion and delusions [26, 43, 44] which would not be predicted by a defence account. Even the presence of a hypothesised defensive externalising attributional style in individuals with persecutory delusions is a topic of debate [67]. Parsimonious explanations of negative emotion and paranoia do not need to evoke defences. In our view, the Poor Me/Bad Me theory has helpfully highlighted one neglected aspect of the content of paranoia, i.e. people may feel they deserve to be harmed, and this is a cognition closely linked to levels of depression. As depression fluctuates so will the ideas about deserving harm, as has been reported [126]; this does not, however, indicate distinct categories. There are other aspects of the content of delusions (e.g. the individual’s perceived degree of control over the situation, ideas about the power of the persecutor) [1] that may also be important in determining or be determined by emotional responses, and, equally, if dichotomised would likely lead to differences in presentation, but this would not indicate discrete subtypes. We endorse the value of identifying emotional correlates of paranoia, but see no convincing empirical evidence of distinct paranoia subtypes, and, indeed, there is evidence against such a view.

### New routes

We have focussed the review upon areas which have received, to some degree, repeated empirical scrutiny. There is, however, much that remains to be investigated in this important area both for individuals’ psychological health and the understanding of the broader issue of social cohesion. Extension of the work into developmental studies of mistrust in children would be especially valuable [16, 127]. This could be linked to the understanding of the metacognitive beliefs that support the adoption of paranoid beliefs [128]. There is initial promising work to take forward examining paranoia in relation to stress [129–131] and emotion regulation difficulties [132, 133]. There are interesting connections between PTSD and paranoia [30, 134, 135] and related innovations in the treatment of psychosis [136]. There is much research relating childhood trauma and abuse to psychosis; while trauma, especially sexual abuse, has been linked to hallucinations, some recently emerging evidence links childhood neglect (such as being placed in care) specifically to paranoia [60, 137]. The precise role of imagery in paranoid fears is yet to be determined [138, 139]. How paranoia affects social functioning [140, 141], what leads to acting on delusions [142], and what are the societal factors that increase paranoia [143] are important topics for future research. Much of our work has been inspired by listening to patients and trying out strategies during psychological therapy, as indicated in the personal accounts used throughout this review. Systematic investigations of patients’ views are likely to be very informative [27, 144]. How paranoia differs in causal factors from anxiety, depression, grandiosity and other related psychological problems remains to be determined [75, 93]. It is notable that the specific neurobiology of delusions and related processing has been neglected [145], while there needs to be a concerted effort to determine the effects at the individual level on psychological processing of known social factors increasing paranoia. Given the clear evidence of advancements in understanding, arguably the greatest focus needs to be on translation into more efficacious treatments and self-management. In this work, the potential benefits of incorporating self-help [146] and technological innovations such as mobile phones [147], experience sampling methodology [148] and virtual reality [149] remains to be determined. In the future, we hope to see many more robust treatment trials focussed specifically upon patients reporting persecutory delusions.
